# Trust and professional relationships drive participation in passive cattle health surveillance among Dutch farmers and veterinarians

**DOI:** 10.3389/fvets.2026.1855833

**Published:** 2026-07-23

**Authors:** Imke Vredenberg, Gerdien van Schaik, Wim H. M. van der Poel, Arjan Stegeman, Jasmeet Kaler

**Affiliations:** 1 Department of Population Health Sciences, Faculty of Veterinary Medicine, Utrecht University, Utrecht, Netherlands; 2 Wageningen Bioveterinary Research, Wageningen University and Research, Lelystad, Netherlands; 3 School of Veterinary Medicine and Science, University of Nottingham, Sutton Bonington Campus, Sutton Bonington, United Kingdom

**Keywords:** cattle, COM-B, health, psychosocial factors, surveillance

## Abstract

Active involvement of farmers and veterinarians is crucial for the effectiveness of passive surveillance. This study aimed to identify the psychosocial factors influencing farmers' and veterinarians' decisions to contact a veterinary helpdesk or the competent authorities regarding cattle health concerns in the Netherlands. We used the COM-B model: for any Behavior (B) to occur, an individual must have the Capability (C), Opportunity (O), and Motivation (M) to perform it. Two separate surveys (for farmers or veterinarians) with Likert-scale statements informed by the COM-B behavioral framework were conducted. For a hypothetical herd of 100 cows, respondents were asked to indicate the weekly mortality threshold at which they thought they would contact a veterinarian, the helpdesk, or the competent authority. In total, 85 farmers and 63 veterinarians completed the survey. Farmers reported they intended to contact their veterinarian at 2.3 cattle deaths per week. Veterinarians indicated they would contact the helpdesk at 3.5 and the competent authority at 5.6 cattle deaths per week. An Exploratory Factor Analysis (EFA) of the farmer responses identified four psychosocial factors: “Farmer-veterinarian relationship,” “Trust in institution,” “Trust in peers and other professionals” and “Confidence.” Regression models were used to examine the association between these psychological factors and reported helpdesk contact and stated mortality thresholds. The first three factors indicated that relational and trust-based factors appear to be important correlates of intended reporting behavior. Despite some variation in responses about the competent authority, no major barriers to engage in passive surveillance were identified. The small sample size and potential selection bias limit the generalizability of the results. Nevertheless, trust-based, low-threshold surveillance systems may improve early detection of animal threats through timely reporting.

## Introduction

Animal health surveillance can contribute to the health and welfare of farm animals through early detection of infectious diseases, allowing timely implementation of measures to prevent disease transmission. In EU countries, surveillance systems for emerging diseases are mandatory under the animal health law (1). Globally, WOAH member countries are required to report notifiable and emerging diseases and are encouraged to provide other relevant information (2).

Surveillance systems comprise several components in which data can be collected in an active or passive way. In the case of active surveillance, data collection is initiated by the investigator (3, 4). Passive surveillance is defined as observer-initiated provision of animal health related data or the use of existing data for surveillance purposes. Decisions about whether information (e.g., clinical signs or mortality) is provided, and what information is provided from which animals are made by the observer (3, 4). Passive surveillance is generally considered most effective, as farmers observe their animals frequently. However, its effectiveness relies on the manifestation of the disease and the timely and complete reporting of relevant animal health information and potential disease outbreaks to the competent authorities. Under-reporting can delay disease detection, thereby increasing the risk of widespread transmission and larger outbreaks.

Veterinarians and farmers are legally required to report suspicions of notifiable diseases and, more broadly, unusual events or signs that may indicate an emerging disease or significant animal health risk. Despite this legal obligation, reporting ultimately depends on individual decision-making in practice, meaning that psychosocial and logistical factors influencing their decision on whether or not to report play an important role in the effectiveness of passive surveillance. Several studies have examined farmers' decision-making around disease control, including one specifically on passive surveillance of notifiable diseases (5). Economic considerations, perceived risk, knowledge and perceived control, as well as incentives, emotions and normative beliefs have previously been reported as important factors in decision-making (5–10). Studies on decision-making in related contexts, such as antimicrobial usage, have identified cost-benefit considerations, normative beliefs, perceived risk, knowledge and perceived social pressure as important drivers of veterinarians' decision-making (11–15). Together, this evidence suggests that farmers and veterinarians share common behavioral determinants influencing their decisions on disease control and reporting. However, research specifically addressing passive surveillance, particularly beyond the obligatory reporting of notifiable diseases, remains limited. Moreover, the relative importance of different behavioral domains, particularly the role of social and relational factors, remains insufficiently understood.

The use of comprehensive behavioral frameworks provides a structured approach to studying and understanding behavior and decision-making processes (16–18). In psychology, the COM-B framework describes behavior (B) by three elements: capability (C), opportunity (O), and motivation (M), which can be used as targets for behavioral change (19). Each element consists of two sub elements. Capability includes psychological and physical aspects, opportunity includes physical and social aspects, and motivation includes automatic and reflective drivers. Examples of psychological capability include knowledge and mental capacity, while physical capability encompasses skills needed to perform the behavior. Opportunity encompasses external factors that facilitate or hinder a target behavior, either in a physical sense (e.g., time, location, resources) or in a social sense (e.g., social pressure, professional relationship, influence of friends and family). Motivation refers to the internal drivers that influence the individual to perform the target behavior, which can be automatic (e.g., impulsions, habits) or reflective, involving conscious planning and evaluation. Despite the utility of theoretical frameworks like the Theory of Planned Behavior or COM-B, studies focusing on farmer and veterinarian behavior often lack their application (6). Applying such frameworks is essential to disentangle which behavioral components are most influential in specific contexts, such as passive surveillance.

Dutch passive surveillance consists of an obligatory and voluntary part. In case of a suspicion of a notifiable disease, the farmer and veterinarian are legally obliged to report their suspicion to the competent authority, the Dutch Food and Consumer Product Safety Authority (NVWA). For other animal health-related cases or questions, farmers and veterinarians can contact the veterinary helpdesk of Royal GD (Gezondheidsdienst voor Dieren, Deventer, Netherlands). The helpdesk is called “GD Veekijker,” in Dutch “Vee” meaning livestock and “kijker” meaning watcher. Since 2002, GD has been commissioned to manage the Dutch voluntary animal health surveillance system by the Ministry of Agriculture and the cattle industry (20). As part of this system, farmers can submit cattle for postmortem investigation to GD to gain insight into the cause of disease and death at a subsidized rate of about €100–150. Upon contacting the helpdesk or submitting an animal for postmortem, farmers receive diagnostic results and free advice to address the health issue, while simultaneously contributing data used for surveillance purposes. The helpdesk and the postmortem examinations play a crucial role in the passive surveillance system, which extends beyond notifiable diseases (20). The willingness of farmers and veterinarians to share information is crucial for the effectiveness of this surveillance component, ensuring early detection and monitoring of emerging animal health threats.

This study aims to identify the psychosocial factors influencing farmers' and veterinarians' decisions to report disease suspicions to authorities or to consult the help desk for cattle health issues. The COM-B model was applied to explore underlying drivers of reporting behavior and barriers to participation, with the objective of identifying which behavioral components are most influential in shaping reporting decisions. In the Dutch context, these findings are relevant for improving passive surveillance. Given that passive surveillance is the cornerstone of emerging disease detection in many countries, the behavioral mechanisms identified in this study, particularly the role of trust and professional relationships, are likely to have a broader relevance for surveillance systems in comparable contexts.

## Material and methods

### Study design and survey development

The survey items were a priori developed based on the components of the COM-B framework and consisted of two separate surveys, one for cattle farmers and one for veterinarians. The target behavior in this study was contacting the veterinary helpdesk or the competent authority in response to cattle health problems related to infectious diseases. The survey for farmers included 38 statements covering all components of the COM-B framework, assessed using a 5-level Likert scale. Additionally, the survey collected demographic and farm-related information, including respondent characteristics (e.g., gender, age) and farm characteristics (e.g., farm type, farm size), as well as slider questions for cattle mortality thresholds at which farmers would decide to contact their veterinarian or GD helpdesk. The survey concluded with an open-ended text box for additional comments. The complete survey including the mapping of the statements to the COM-B framework can be found in Appendix A1.

The farmer and veterinarian questionnaires were designed to be conceptually aligned while accounting for differences in professional roles and contexts. For example, questions regarding peer influence referred to fellow farmers in the farmer survey and to fellow veterinarians in the veterinarian survey (Appendix A2). The survey consisted of seven general questions about the veterinarian and veterinary practice, 41 statements on a 5-level Likert scale, and slider questions indicating at which level of mortality (i) the veterinarian is contacted by the farmer, (ii) the veterinarian contacts GD helpdesk, and (iii) the competent authority is contacted.

Both the farmer and veterinarian surveys began with a brief introduction, followed by a confidentiality and consent statement. Participants were required to provide informed consent by ticking a box before proceeding with the survey. To ensure clarity and usability, both surveys were pilot tested on a small sample (*n* = 5). Participants were asked to complete the questionnaire and subsequently provide feedback on the wording, interpretation, and answerability of the questions. Based on the feedback received, only minor textual revisions were made to improve clarity and wording, and definitions were added for some phrases. The original survey was developed in Dutch using Qualtrics XM (Provo, Utah, United States). The study was approved by the Science-Geoscience Ethics Review Board (SG ERB) of Utrecht University (ERB Review Vet R-25.011).

### Survey distribution

**Figure 1:**
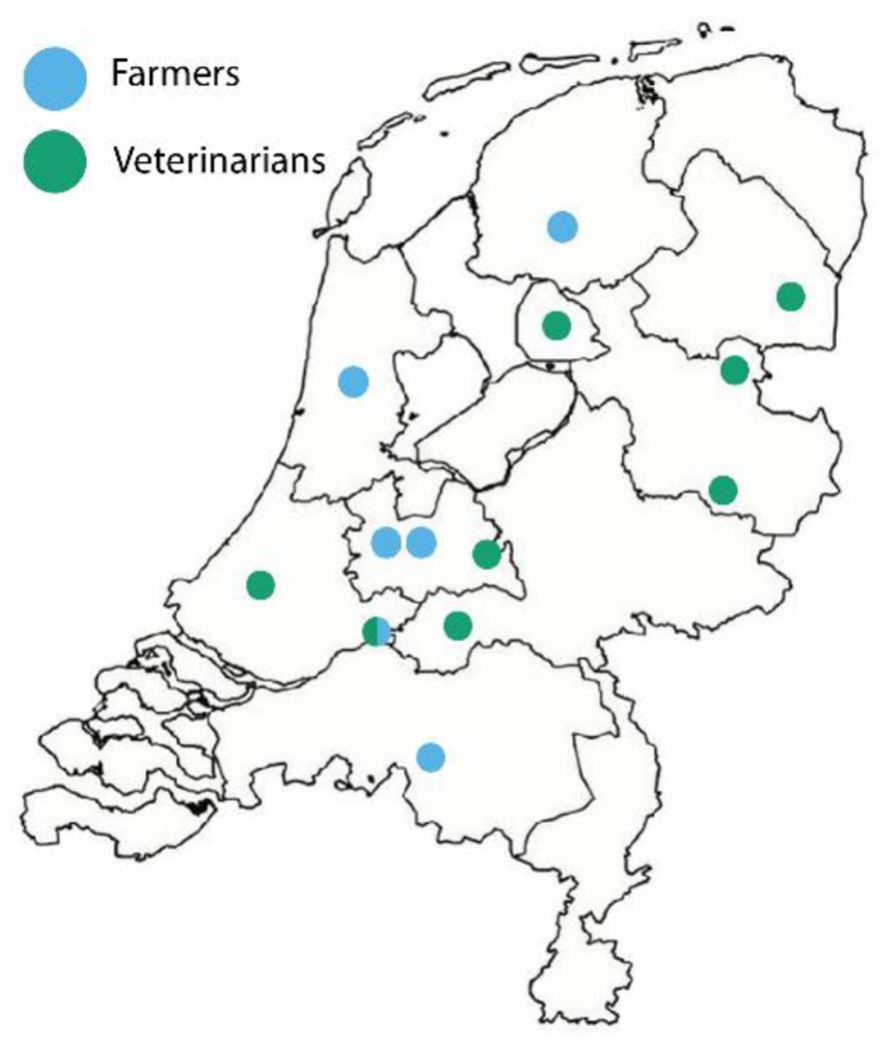
Geographical distribution of the survey collection locations in the Netherlands. Blue dots represent the locations for veterinarians and green for farmers. At the shared dot both surveys were taken.

All respondents were provided personal links to the electronic surveys. The surveys for farmers were collected at a three-day agricultural fair for the cattle sector (*N* = 35) and during seven meetings of a farmer association, held across the country (*N* = 105, Figure 1). The topics addressed in these meetings were not related to this study, and farmers were not informed in advance about the researcher's presence. The meetings primarily targeted dairy farmers. A total of 140 farmers started the survey, of whom 85 (60.7%) completed it fully.

The surveys for veterinarians were collected at six intervision-meetings (i.e. meetings to discuss work-related issues with peers) across the country (*N* = 67, Figure 1) and at the agricultural fair (*N* = 7). The survey was also published on the website of the Dutch veterinary association and distributed by email to veterinary practices affiliated with the Faculty of Veterinary Medicine at Utrecht University (*N* = 9). These nine veterinary practices could be approached directly through existing professional networks and were not selected based on their involvement in surveillance activities or any other characteristics relevant to the study. A total of 83 veterinarians started the survey, of whom 63 (75.9%) completed it fully.

### Data processing

After collecting the surveys, eight questions for farmers and five for veterinarians were recoded. These questions were originally framed in a negative way in relation to the target behavior, contacting the veterinary helpdesk or the competent authority. For example, “Contacting GD helpdesk is time consuming.”

The variable “Did you ever have contact with GD” contained the answers “Yes,” “No” and “Don't know/ don't want to say.” Because of the low number of people selecting “Don't know/ don't want to say” (*n* = 6), the category was recoded as “No” to maintain sufficient statistical power for analysis. Additionally, one participant did not answer the question and was initially coded as “Don't know/ don't want to tell,” but was later recoded as “No.” The age categories in the survey were up to 29 years, 30–39, 40–49, 50–59 and 60 years or older. All participants aged 60 and older had previously contacted GD. Due to the lack of variability within this group we merged this age category with the 50–59 years old participants, creating a new 50 years and older category, which was subsequently used in the regression models.

Participants were asked about the weekly mortality in a hypothetical herd of 100 cattle at which they would call their veterinarian or the veterinary helpdesk. For dairy herds, the question was asked for calves (< 1 year) and adult cattle (>1 year) separately. Veal calf farmers were only asked about calf mortality. Because 98% of respondents were dairy farmers, we could not meaningfully distinguish mortality by farm type.

### Data analysis

Exploratory Factor Analysis (EFA) was performed on the data of both farmers and veterinarians. However, due to the small sample size, the EFA on the survey results of the veterinarians did not provide robust results. Therefore, only descriptive statistics are presented for the veterinarian's survey. Exploratory Factor Analysis is a technique to find latent (unmeasured) variables based on the measured variables (21). Latent variables are often hard to measure directly, but by asking related questions, they can be identified using EFA. The EFA groups related questions into factors, which serve as the latent variables. For example, the response on several statements that indicate the trust in an institution or the willingness to execute a certain behavior load on the same factor. Directly asking about trust or behavior will more likely result in socially acceptable answers. We used the Oblimin rotation method for the EFA, which allows latent variables to be correlated with each other (22). Each respondent received a factor score based on their responses to the questions that loaded onto the factor. A factor loading less than 0.3 was considered too low to be meaningful, given that it indicates that the variable shares less than 10% of its variance with the latent factor.

We used complete cases for the questions of the COM-B model to identify latent variables. Initially, a scree plot suggested retaining five factors. Items (questions) with a factor loading less than 0.3 were excluded. Cross-loading items (i.e., items loading on multiple factors) were removed if the difference in loading between items was less than 0.3. After each removal of items, the EFA was rerun on the remaining items. This iterative process continued until all retained factors met the predefined criteria i) all items had a factor loading of at least 0.3, ii) a cross-loading of more than 0.3 with other items, iii) each factor contained at least three items and iv) all items had a communality score higher than 0.25. Communality and uniqueness indicate how much variation of an item is explained by the factor (communality) vs. how much is unique to the item (uniqueness) (23, 24). Scores of uniqueness and communality range from 0 to 1 and sum to 1. The factor labels were assigned based on the content and conceptual similarity of the survey items with the highest loadings on each factor. The final extracted factors were used as independent variables in a logistic regression model with the question “Did you ever contact the veterinary helpdesk?” as dependent binary (yes or no) variable and logit link-function. A Poisson regression model (the number of dead animals with a log link-function), was used to determine the association between the mortality levels of cattle and calves in a hypothetical herd of 100 cows at which farmers reported to contact their veterinarian or the helpdesk and the factors from the EFA. In addition to the four factors from the EFA, initially farm location, farmer's age category, gender, herd size, and farm type, were included in the full models. The models were fitted using the GLM function (MASS, version 7.3–60) with backwards variable selection at *P* < 0.05 based on the model's AIC using the drop1 (stats, version 4.3.1) function in R (version 4.3.1). When the estimates of variables in the model changed with >25% a variable was considered a confounder and was retained in the model. To check robustness, a forward selection was carried out by adding one by one the variable with the lowest *P*-value to the model till all variables in the model had a *P* < 0.05 or were a confounder. Two-way interactions were tested and none were significant. Diagnostic checks indicated no evidence of substantial overdispersion; therefore, the Poisson model assumptions were considered adequate.

## Results

### Description of farmers

**Figure 2:**
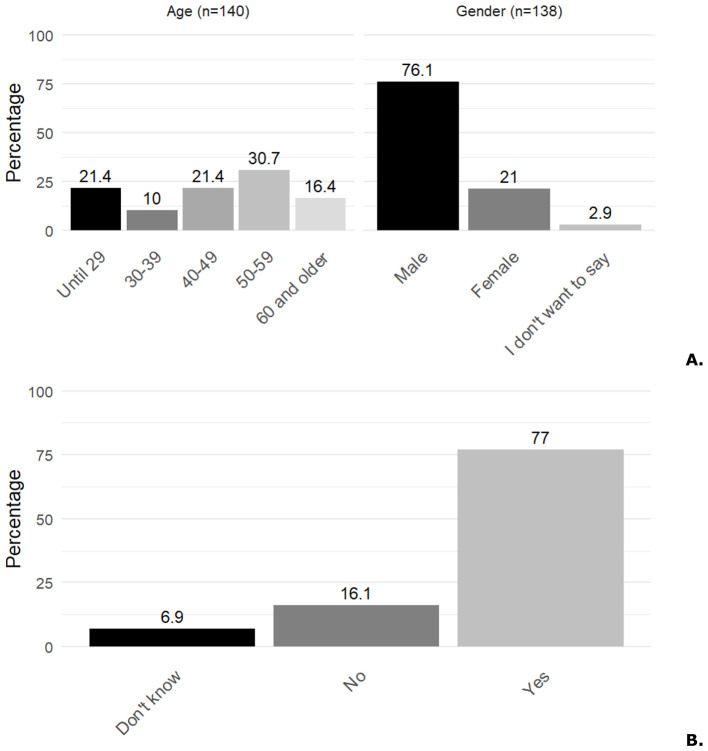
**(A)** Distribution of age and gender of farmers participating in the COM-B survey on reporting behavior in the Netherlands. **(B)** Answers provided by Dutch farmers whether they had ever contacted the veterinary helpdesk of GD.

Between November 2023 and April 2024, a total of 140 surveys were started. Of these, 85 surveys were fully completed, while 87 respondents completed at least the Likert scale questions. The largest age group among respondents was 50 to 59 years old, and most participants were male (*n* = 105, 76.1%) (Figure 2A). Dairy farming was identified as the main farm type by 96.2% of the respondents, while the remaining participants selected other primary farm types, such as veal, or youngstock rearing. Additionally, 77% of the farmers (*n* = 108) reported having contacted the veterinary helpdesk at least once (Figure 2B).

**Figure 3:**
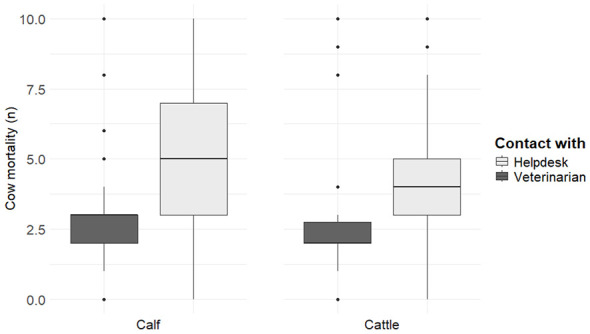
Distribution of the weekly farmer' stated cattle mortality at which Dutch farmers (n = 85) reported to contact their veterinarian (left box) or the veterinary helpdesk of GD (right box) for calves and cattle, respectively.

Participants were asked to indicate the weekly cattle and calf mortality, based on a farm size of hundred cattle, at which they would contact their veterinarian or the veterinary helpdesk. Farmers reported that they would contact their veterinarian at mean of 2.8 (median = 3) calf deaths or 2.3 (median = 2) cattle deaths per week (Figure 3). Farmers reported that the helpdesk would be contacted at mortality levels of on average 5.0 (median = 5) calf deaths and 4.2 (median = 4) cattle deaths per week (Figure 3). Greater variability was observed in reported helpdesk contact thresholds for both cattle (SD_Cattle_ = 2.8) and calves (SD_Calf_ = 2.9) compared to reported contact with their veterinarian (SD_Cattle_ = 1.7, SD_calf_ = 1.5).

The top three statements, on a 5-level Likert scale, with the highest standard deviation and the top three with the lowest standard deviation were determined (Table 1). On average, the three statements with the highest variability were answered around the neutral category (neither agree nor disagree). The statement “The professional relation with my veterinarian influences my decision to call GD Veekijker” showed a slight tendency toward agreement (mean = 3.42, median = 4), while “I don't like it when my veterinarian reports a suspicion on my farm to the NVWA” leaned toward disagreement (mean = 2.55, median = 3). The three statements with lowest variation had a mean score above 4 and median of 5, indicating strong agreement among participants. Additionally, the statements “Discussing clinical symptoms with my veterinarian is useful” and “I feel good when I discussed a case with my veterinarian” also showed low variability (SD = 0.73).

**Table 1:** The top three statements with the highest and lowest variation in answers given by Dutch farmers participating in a survey on reporting behavior (n = 85).

Proposition top 3 highest SD	Mean	SD	Median
The professional relation with my veterinarian influences my decision to call GD Veekijker	3.42	1.19	4
I know which clinical signs I can discuss with GD Veekijker	3.20	1.15	3
I don t like it when my veterinarian reports a suspicion on my farm to the NVWA	2.55	1.14	3
Proposition top 3 lowest SD			
I trust the expertise of my veterinarian on infectious disease	4.52	0.66	5
I am willing to follow the advice of my veterinarian on infectious disease	4.41	0.67	5
I know for which animal health problems I should involve the veterinarian	4.43	0.73	5

The statements were based on the COM-B model and on a 5-level Likert scale (1 = totally disagree to 5 totally agree).

### Factor analysis farmers

Four factors were identified: “Farmer-veterinarian relationship”, “Trust in institution”, “Trust in peers and other professionals” and “Confidence” (Table 2). A fifth factor dropped out because only two items loaded on that factor. The factor labels were assigned based on the content and conceptual similarity of the survey items with the highest loadings on each factor. For example, the items loading on the factor labeled “Farmer-veterinarian relationship” all related to trust in, communication with, and perceived value of interactions with the veterinarian. This factor reflects the quality of the relationship and interaction between farmers and veterinarians. The second factor “Trust in Institution” contains perceptions of and trust in the veterinary helpdesk. The third factor includes three statements about fellow farmers and non-veterinary professionals. The last factor reflects the farmer's confidence in their own knowledge and abilities. The final item in the confidence factor was negatively loaded, meaning that it should be interpreted in reverse, farmers with higher scores are less inclined to prefer their veterinarians contacting the veterinary helpdesk on their behalf.

**Table 2:** Exploratory factor analysis results from a COM-B survey with 85 Dutch farmers on reporting behavior. Four factors were identified and named.

Factor name	Survey question	Factor loading	Communality
Farmer-veterinarian relationship	I trust the expertise of my veterinarian on infectious disease	0.75	0.54
	I am willing to follow the advice of my veterinarian on infectious disease	0.69	0.57
	I feel free to discuss a suspicion with my veterinarian	0.67	0.53
	The information I receive from my veterinarian is useful	0.59	0.41
	Discussing clinical symptoms with my veterinarian is useful	0.59	0.36
	I know for which animal health problems I should involve the veterinarian in	0.58	0.40
	I feel good when I discussed a case my veterinarian	0.50	0.37
Trust in institution	I feel free to discuss a suspicion with GD Veekijker	0.88	0.79
	I consider GD Veekijker a safe place to discuss animal health problems	0.78	0.62
	My fellow farmers stimulate me to contact GD Veekijker in case of animal health problems	0.51	0.34
	I stimulate my veterinarian to contact GD Veekijker in case of animal health problems	0.48	0.34
	My veterinarian stimulates me to contact GD Veekijker in case of animal health problems	0.44	0.27
	I know that I can call GD Veekijker if I want to discuss an animal health problem	0.36	0.32
	GD Veekijker adds to the detection of emerging infectious diseases	0.34	0.32
Trust in peers and other professionals	I trust the expertise of professionals entering my farm other than my veterinarian on infectious disease	0.63	0.42
	Discussing an animal health problem with fellow farmers gives me a good feeling	0.54	0.32
	I feel free to discuss a suspicion with my fellow farmers	0.42	0.28
Confidence	I feel secure in the event of an apparent outbreak of an infectious disease at my farm	0.72	0.59
	I don't worry about introduction of emerging disease in the Netherlands	0.52	0.30
	I want my veterinarian to contact GD Veekijker	−0.50	0.56

The factor loading and communality are provided for the statements which are part of the four factors.

Based on the responses to the Likert scale statements and the EFA results, individual factor scores were calculated. Table 3 shows the correlation between factors. “Farmers-veterinarian relationship” and “Trust in institutions” was the only statistically significant correlation (*r* = 0.30).

**Table 3:** The correlation coefficients between the four factors of the Exploratory Factor Analysis (EFA) on a COM-B survey among Dutch farmers (*N* = 85) about reporting behavior in the Netherlands.

Correlation coefficients	Farmer-veterinarian relationship	Trust in institutions	Trust in peers and other professionals	Confidence
Farmer-veterinarian relationship	1	0.30^*^	0.16	−0.29
Trust in institutions		1	0.22	−0.06
Trust in peers and other professionals			1	−0.09
Confidence				1

^*^Significant *p* < 0.05.

### Psychological factors associated with helpdesk contact by farmers

Farmers with a one-point higher score on the factor “Trust in institution” had a 2.95 (95% CI: 1.52–6.73) higher odds of reporting ever having contacted the helpdesk (Table 4). Farmers in the youngest age groups had a 0.11 (95% CI: 0.02–0.52) lower odds of reporting ever having contacted the helpdesk compared to the 40 to 49 year old participants. The age group 30 to 39 (OR = 1.04, 95% CI: 0.13–11.35) and older than 50 (OR = 3.21, 95% CI: 0.67–17.02) had a higher odds of reporting helpdesk contact, however the 95% CI were extremely wide and these differences were not statistically significant compared to the 40- to 49-year-olds. No other variables were significantly associated with reporting helpdesk contact.

**Table 4:** The associations between reporting ever contacting the veterinary helpdesk of GD by farmers and farmer scores on factors obtained from a factor analysis on a COM-B survey about reporting behavior in the Netherlands.

Variable	Categories	β	OR	CI 95%
Intercept		1.48		1.56–15.61^*^
Trust in institution		1.08	2.95	1.52–6.73^*^
Age	−29	−2.24	0.11	0.02–0.52^*^
	30–39	0.03	1.04	0.13–11.35
	40–49	Ref.	Ref.	Ref.
	50+	1.17	3.21	0.67–17.02

^*^Significant *p* < 0.05.

### Psychological factors associated with mortality thresholds

Associations between farmer characteristics and weekly mortality thresholds at which farmers said that they would seek help were determined separately for cattle and calves (Table 5). Farmers that scored one-point higher in “Farmer-veterinarian relationship” had a 0.85 (CI 95%: 0.74–0.98) times lower cattle mortality threshold at which farmers reported to contact a veterinarian. Similarly, farmers with higher “Trust in institution” scores were associated with lower mortality thresholds at which farmers reported to contact the helpdesk (IRR = 0.87; CI 95%:0.78–0.96). For calf mortality, “Trust in peers and other professionals” and gender were significantly associated with reporting helpdesk contact. Female farmers reported to contact the helpdesk at a lower mortality threshold (IRR = 0.59: CI 95%: 0.42–0.82) compared to male farmers. A one-point higher score in “Trust in peers and other professionals” resulted in a 0.88 (CI 95%: 0.77–0.99) times lower calf mortality threshold at which farmers reported to contact the helpdesk. No significant associations were identified between reporting to contact the veterinarian and stated calf mortality.

**Table 5:** The associations between the stated number of dead animals at which farmers (n = 85) report to contact their veterinarian or the veterinary helpdesk and farmer characteristics and location where the survey was taken in the Netherlands.

		Veterinarian				Helpdesk			
Variable	Categories	β	IR	IRR	CI 95%	β	IR	IRR	CI 95%
Cattle									
Intercept		0.85	2.34		2.03–2.69^*^	1.37	3.96		3.10–4.93^*^
Farmer-veterinarian relationship		−0.16		0.85	0.74–0.98^*^				
Trust in institution						−0.14		0.87	0.78–0.96^*^
Calf									
Intercept		1.04	2.83		2.48–3.21^*^	1.60	4.94		3.89–6.10^*^
Trust in peers and other professionals						−0.13		0.88	0.77–0.99^*^
Gender	Female					−0.52 0.25		0.59 1.28	0.42–0.82^*^ 0.70–2.19
	Unknown								

^*^Significant *p* < 0.05. The incidence rate (IR) is the count of death cattle at which the veterinarian or helpdesk is contacted. The incidence rate ratio (IRR) is the relative increase or decrease of that count.

### Description of veterinarians

**Figure 4:**
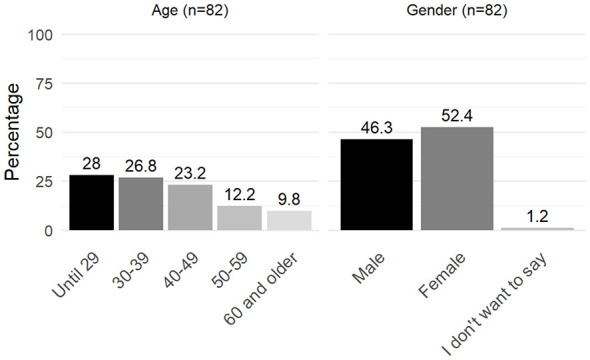
Distribution of age and gender of veterinarians (*n* = 82) participating in a COM-B survey about reporting behavior in the Netherlands.

A total of 82 veterinarians started the survey, of whom 63 fully completed it, while 66 completed at least the Likert scale statements (Figure 4). More than half of the veterinarians were under the age of 39 (*n* = 45), while 9.8% were 60 or older (*n* = 8). Gender distribution was more balanced compared to the farmers, with 52.4% female and 46.3% male participants, and 1.2% not disclosing their gender (*n* = 82).

**Figure 5:**
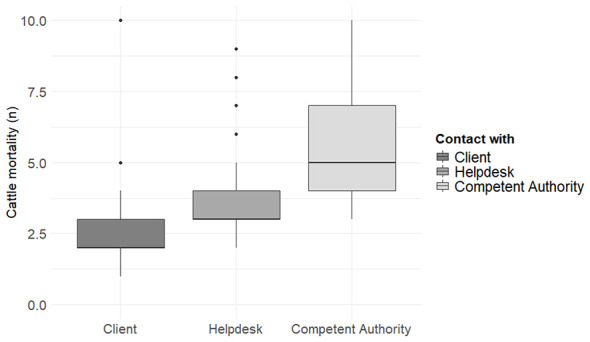
Distribution of the weekly cattle mortality at which veterinarians (*n* = 63) estimate when farmers would call, when veterinarians would call the veterinary helpdesk and the competent authority in the Netherlands.

Veterinarians estimated that farmers would contact them at on average 2.5 dead cattle within a week (median = 2) assuming a herd size of 100 cows (Figure 5). They reported that they would contact the helpdesk at on average 3.5 (median = 3) cattle deaths in a week and the competent authority at 5.6 (median = 5) cattle deaths. Variability was highest for contacting the competent authority (SD = 2.00) compared to contacting the helpdesk (SD = 1.48) or being contacted by farmers.

The three statements with the highest variation in Likert scale scores represented different COM-B domains (Table 6). Two statements showed responses leaning toward disagreement. “I know that I can email GD Veekijker if I want to discuss a case” (mean = 2.75) and “I take the legal consequences of not reporting to the competent authority into account” (mean = 2.95). The statements with the lowest variation (SD = 0.61–0.56) were all strongly agreed upon, indicating high consensus among respondents.

**Table 6:** The top three statements with the highest and lowest variation in answers given by the veterinary participants (n = 63) of the survey about reporting behavior in the Netherlands. The statements were based on the COM-B model and on a 5-level Likert scale (1 = totally disagree to 5 totally agree).

Proposition top 3 highest SD	Mean	SD	Median
I know that I can email GD Veekijker if I want to discuss a case	2.75	1.34	3
It is part of my routine to call GD Veekijker in a complex case	3.38	1.25	4
I take the legal consequences of not reporting to the competent authority into account	2.95	1.25	3
Proposition top 3 lowest SD			
I know that I can call GD Veekijker if I want to discuss a case	4.74	0.56	5
My clients are willing to follow my advice on infectious disease	4.03	0.61	4
I have the knowledge to recognize when infectious disease in cattle needs to be reported to the competent authority	4.17	0.61	4

## Discussion

This study provides insight into psychosocial factors underlying farmers' and veterinarians' reported participation in passive cattle health surveillance, with a specific focus on decisions to contact the veterinary helpdesk or competent authorities. Using the COM-B framework, we demonstrated that social opportunity, particularly trust and professional relationships, are dominant drivers of stated reporting behavior, whereas major structural or motivational barriers appear limited in the Dutch context. An important contribution of this study is the application of a behavioral framework to passive surveillance, an area where empirical evidence remains scarce. While previous research has identified economic, cognitive, and normative drivers of disease control behavior (6–8, 10), fewer studies have systematically examined reporting behavior beyond notifiable diseases (5), which is important for newly emerging diseases. Our findings indicate that decision-making in passive surveillance is strongly embedded in social and relational contexts, rather than being primarily constrained by knowledge or capacity. This has important implications for how surveillance systems are designed and supported.

Three factors, “farmer-veterinarian relationship,” “trust in institution,” and “trust in peers and other professionals,” emerged as central correlates of farmers' reported behavioral intentions. All three map onto the social opportunity component of the COM-B model (19), highlighting the importance of interpersonal trust, social norms, and institutional confidence. This aligns with previous work demonstrating that trust-based relationships are critical for the uptake of disease control measures, such as bovine viral diarrhea programs (25), but extends this insight to the domain of surveillance and reporting.

The “farmer-veterinarian relationship” factor was associated with farmers reporting earlier contact with veterinarians at lower mortality thresholds, suggesting that strong, trust-based relationships facilitate more proactive engagement. This is in line with previous studies demonstrating that effective veterinary-farmer relationships are built through continuity, regular interaction, and shared understanding of goals and constraints (26, 27). Such relationships have been shown to influence the uptake of biosecurity measures and disease management practices (9, 27–29). From a behavioral perspective, this suggests that improving passive surveillance should not focus solely on increasing knowledge, but also on strengthening communication and collaboration, for example through approaches such as Motivational Interviewing.

“Trust in institutions” was associated with an increased likelihood and timeliness of reporting to contact the veterinary helpdesk. This finding is highly relevant from a surveillance system design perspective. In contrast to governmental authorities, GD functions as an independent, sector-embedded intermediary, providing diagnostic services and advisory support without automatically triggering regulatory consequences. This structure likely reduces perceived risk associated with reporting and lowers the threshold for engagement. Similar challenges related to institutional trust have been described in other contexts, where farmers expressed distrust in governmental bodies due to perceived lack of expertise or inconsistent communication (30, 31). Our findings suggest that institutional design, specifically the presence of a trusted, low-threshold interface, may be a critical enabler of effective passive surveillance.

The factor “trust in peers and other professionals” was associated with farmers stating earlier reporting at lower calf mortality thresholds, highlighting the role of peer networks and social norms. Disease reporting is not only a technical decision but also a socially embedded act, often influenced by concerns about reputation and stigma. Prior research has shown that farmers may fear being perceived as responsible for the introduction or spread of infectious diseases, potentially affecting their identity as a “good farmer” (32–34). Strong peer relationships may help mitigate these concerns and support more open communication about animal health issues.

Interestingly, the study did not identify substantial barriers that would undermine the functioning of passive surveillance. This contrasts with previous literature emphasizing underreporting and behavioral constraints (5). One possible explanation is the specific Dutch context, where surveillance is supported by well-established infrastructure and institutional arrangements (4, 20). This suggests that passive surveillance systems can function effectively when embedded in trusted professional networks and supported by accessible, non-punitive reporting structures. However, the findings may also be due to selection bias of a group willing to participate in the survey of which many had contacted the veterinary helpdesk.

Several demographic factors were associated with farmers stated reporting behavior to the veterinary helpdesk. Age was positively associated with reporting to ever having contacted the veterinary helpdesk, which is expected as older farmers have typically been in the profession longer, increasing their likelihood of encountering animal health problems, and consequently their chances of contacting the helpdesk. Gender also played a role, with female farmers reporting contact at a lower calf mortality level compared to male farmers. This may reflect gendered roles on farms, where women are often primarily responsible for calf care (35).

While farmers and veterinarians reported broadly similar thresholds for contacting each other and the veterinary helpdesk, greater variability was found in decisions to contact the competent authority. This variability likely reflects ambiguity in regulatory thresholds and competing professional considerations. The absence of a clear definition of excess mortality in the animal health legislation, may lead to different interpretations of reporting requirements. Additionally, veterinarians may experience tension between their advisory role and their legal obligations, particularly when reporting could have significant economic or social consequences for farmers, such as the risk of temporary farm closures, which may discourage reporting to the competent authority. Similar role conflicts have been described in other areas of veterinary practice, such as antimicrobial stewardship (13–15).

Several limitations should be considered when interpreting these findings. First, the study relies on self-reported behavior, which may be influenced by social desirability bias and may not fully reflect actual practices (36). In reality, mortality thresholds may be higher and contact frequencies lower than reported. Second, the sampling strategy likely resulted in a selection of relatively engaged and well-connected participants, as farmers were primarily recruited through sector meetings and veterinarians through knowledge exchange sessions. This may have led to an overrepresentation of individuals who are already more positively inclined toward surveillance participation. However, this type of selection bias is more likely to affect estimates of absolute behavior, such as reported contact frequencies or mortality thresholds, than the structure of associations between variables, provided that selection is not jointly related to both the determinants and the outcomes of interest (37). As the primary aim of this study was to identify psychosocial determinants of reporting behavior rather than to estimate population-level reporting rates, the observed associations between factors such as trust and professional relationships and stated reporting behavior remain informative. In addition, this aligns with broader behavioral literature emphasizing the central role of social norms and relationships in decision-making (6, 16).

Additional limitations include the recoding of “don't know/don't want to say” responses as “no,” which may have introduced bias, and the limited sample size for veterinarians, which prevented factor analysis in this group. Survey fatigue and questionnaire length may also have contributed to incomplete responses. The sample size for farmers was sufficient to run an EFA and the results seemed robust and in line with earlier findings. However, the ratio of respondents to variables was fairly low. The required sample size depends on the correlation among the variable, the higher the correlation, the lower the sample size may be (21). The variables within the constructs of the COM-B model are meant to be correlated and the EFA on the farmers survey resulted in four logical and relevant psychosocial factors. However, the results from the EFA should be considered as exploratory and interpreted with some caution. For the veterinarians survey, the sample size was too low for an EFA, not resulting in factors with more than two items.

The absence of major barriers in this sample does not imply that such barriers are absent in general, but rather that they may be effectively mitigated within well-functioning surveillance systems. This supports the interpretation that the Dutch system, with its institutional design and embedded trust structures, may reduce barriers that are more prominent in other contexts. The findings are most directly applicable to surveillance systems in high-income countries with comparable infrastructure and institutional arrangements. In settings where trust in institutions is lower or where reporting is more closely linked to regulatory enforcement, different behavioral dynamics may be expected. Future research should therefore explore how institutional context shapes reporting behavior, for example through qualitative or comparative studies. In those comparative studies, stratified random samples of farmers should be recruited, including those with no prior helpdesk contact, to assess whether the trust-based associations observed here hold in less-engaged populations.

In conclusion, this study indicates that reported participation in passive surveillance is strongly influenced by social and relational factors, particularly trust in institutions and professional relationships. In the Dutch context, no major behavioral barriers were identified that would compromise early detection of emerging diseases. Instead, the findings highlight the importance of maintaining and strengthening trust-based interactions and institutional arrangements that facilitate low-threshold reporting. Future research should further explore the underlying mechanisms of trust and their implications for surveillance system design.

## Data Availability

The datasets presented in this article are not readily available because the participant only gave consent for use of their data in the current study. Requests to access the datasets should be directed to g.vanschaik@uu.nl.
