# Supplementary material for: Trust and professional relationships drive participation in passive cattle health surveillance among Dutch farmers and veterinarians
Source: Front Vet Sci. 2026 Jul 23;13:1855833. doi: 10.3389/fvets.2026.1855833 (PMC13441842; doi:10.3389/fvets.2026.1855833)
Supplement: Supplementary file 1 [file Supplementary_file_1.docx]

**Appendix**

**A1. Survey farmers**

**Farm and farmer characteristics**

- Select your age category.
- Select your gender.
- What are the first two numbers of the postal code of the farm ?
- Select your main farm type.
- How many animals do you have:
  - Dairy farms: How many dairy cattle do you have?
  - Veal calf operations: How many calves do you have?
  - Rearing farm: How many cattle do you have?
  - Suckler farm: How many adult cattle do you have?
  - Miscellaneous: How many cattle do you have?
- Did you ever had contact with GD Veekijker?

**Part 1**

**Statements based on the COM-B framework (5-level Likert scale)**

Capability

*Psychological*

- I know of the existence of the GD Veekijker
- I know for which animal health problems I should involve the veterinarian in.
- I have the knowledge to recognize clinical signs in cattle related to notifiable disease
- I know which clinical signs I can discus with GD Veekijker

*Physical*

- I know that I can email GD Veekijker if I want to discuss an animal health problem
- I know that I can call GD Veekijker if I want to discuss an animal health problem

Opportunity

*Physical*

- Discussing clinical symptoms with my veterinarian is useful
- Contacting GD Veekijker is time consuming
- Discussing clinical symptoms with GD Veekijker is useful
- Contacting my veterinarian is time consuming
- The information I receive from my veterinarian is useful
- The information I receive from GD Veekijker is useful

*Social*

- I feel free to discuss a suspicion with professionals entering my farm other than my veterinarian
- I stimulate my veterinarian to contact GD Veekijker in case of animal health problems
- The professional relation with my veterinarian influences my decision to call GD Veekijker
- I feel free to discuss a suspicion with my veterinarian
- I am willing to follow the advice of my veterinarian on infectious disease
- My fellow farmers stimulate me to contact GD Veekijker in case of animal health problems
- I trust the expertise of my veterinarians on infectious disease
- I want my veterinarian to contact GD Veekijker
- My veterinarian stimulates me to contact GD Veekijker in case of animal health problems
- I feel free to discuss a suspicion with my fellow farmers
- I trust the expertise of professionals entering my farm other than my veterinarian on infectious disease
- I feel free to discuss a suspicion with GD Veekijker
- I don’t like it when my veterinarian reports a suspicion on my farm to the NVWA

Motivation

*Automatic*

- I don't want it to turn out later that I did not report a suspicion
- Discussing an animal health problem with professionals entering my farm other than my veterinarian gives me a good feeling
- I feel good when I discussed a case my veterinarian
- I feel good when I discussed a case with GD Veekijker
- Discussing an animal health problem with fellow farmers gives me a good feeling
- I feel insecure in the event of an apparent outbreak of an infectious disease at a neighbouring farm
- I consider GD Veekijker a safe place to discuss animal health problems
- I worry about introduction of emerging disease in the Netherlands
- I feel insecure in the event of an apparent outbreak of an infectious disease at my farm

*Reflective*

- Reporting to GD Veekijker can have negative consequences for my farm
- I feel responsible for reporting of clinical signs which are related to emerging diseases
- GD Veekijker adds to the detection of emerging infectious diseases
- Reporting to the competent authority can have negative consequences for my farm

**Part 2**

**Estimating cattle and calf mortality thresholds for contact**

At how many death cows within 7 days is it likely that:

- You call your veterinarian
- You call GD Veekijker

At how many death calves within 7 days is it likely that:

- You call your veterinarian
- You call GD Veekijker

**A2. Survey veterinarians**

**Veterinarian and veterinary practice characteristics**

- Select your age category.
- Select your gender.
- What are the first two numbers of the postal code of the veterinary practice ?
- How many veterinarians are employed at your practice?
- How many veterinarians are working on cattle?
- How many farms are affiliated to your practice?
- Is your company part of a corporation?

**Part 1**

**Statements based on the COM-B framework (5-level Likert scale)**

Capability

*Psychological*

- I know of the existence of the GD Veekijker
- I have the knowledge to recognize when infectious disease in cattle needs to be reported to the competent authority.
- I know at which level of mortality I have to report to the competent authority
- I recognize similarities in clinical symptoms between farms related to infectious disease
- I know how to advise farmers about the control of infectious diseases in cattle in my own practice
- I know which clinical signs I can discus with GD Veekijker

*Physical*

- I know how to get in contact with the competent authority to report animal disease
- I know that I can email GD Veekijker if I want to discuss a case
- I know that I can call GD Veekijker if I want to discuss a case

Opportunity

*Physical*

- Contacting GD Veekijker is time consuming
- Discussing clinical symptoms with GD Veekijker is useful
- Contacting the competent authority is time consuming
- The information I receive from GD Veekijker is useful

*Social*

- I feel free to discuss a suspicion with my colleagues from other practices
- My clients stimulate me to contact GD Veekijker in case of animal health problems
- My colleagues stimulates me to contact GD Veekijker in case animal health problems
- I feel pressured to report a suspicion to the competent authority
- I feel free to call GD Veekijker to discuss a suspicion
- My clients opinion influence my decision to call GD Veekijker
- My clients trust my expertise on infectious disease
- My clients want me to contact GD Veekijker
- I feel free to call GD Veekijker to discuss a case
- I take the legal consequences of not reporting to the competent authority into account
- My clients opinion influence my decision to call the competent authority
- I feel free to discuss a suspicion with my colleagues from my practice
- My clients want me to report notifiable disease to the competent authority
- My clients are willing to follow my advice on infectious disease

Motivation

*Automatic*

- I feel insecure in the event of an apparent outbreak of an infectious disease at a farm
- I feel good to report a suspicion to the competent authority
- I feel good when I discussed a case with GD Veekijker
- It is part of my routine to call GD Veekijker in a complex case
- I don't want it to turn out later that I did not report a suspicion
- I consider GD Veekijker a safe place to discuss animal health problems
- I worry about introduction of emerging disease in the Netherlands
- Not reporting a vague suspicion gives me a bad feeling
- Discussing a case with colleagues gives me a good feeling

*Reflective*

- GD Veekijker adds to the detection of emerging infectious diseases
- I feel responsible for reporting of clinical signs which are related to emerging diseases
- Reporting to the Authorities can have negative consequences for my clients
- Reporting to GD Veekijker can have negative consequences for my clients
- I should have more contact with GD Veekijker

**Part 2**

**Estimating cattle and calf mortality thresholds for contact**

Your client has on around 100 lactating cows. At how many death cows within 7 days is it likely that:

- Your client contacts you
- You call GD Veekijker
- You call the CA
